# Enhanced susceptibility to stress and seizures in GAD65 deficient mice

**DOI:** 10.1371/journal.pone.0191794

**Published:** 2018-01-29

**Authors:** Jin Qi, Minjung Kim, Russell Sanchez, Saba M Ziaee, Jhumku D Kohtz, Sookyong Koh

**Affiliations:** 1 Neurobiology Program, Stanley Manne Children’s Research Institute, Chicago, IL United States of America; 2 Department of Pediatrics, Emory University School of Medicine, Atlanta, GA United States of America; 3 Developmental Biology, Stanley Manne Children’s Research Institute, Chicago, IL United States of America; University of Modena and Reggio Emilia, ITALY

## Abstract

Reduced gamma-aminobutyric acid (GABA) inhibition has been implicated in both anxiety and epilepsy. GAD65^-/-^ (NOD/LtJ) mice have significantly decreased basal GABA levels in the brain and a lowered threshold for seizure generation. One fifth of GAD65 ^-/-^ mice experienced stress-induced seizures upon exposure to an open field at 4 weeks of age. In each successive week until 8 weeks of age, the latency to seizures decreased with prior seizure experience. 100% of GAD65^-/-^ mice exhibited stress-induced seizures by the end of 8 weeks. GAD65^-/-^ mice also exhibited marked impairment in open field exploratory behavior and deficits in spatial learning acquisition on a Barnes maze. Anxiety-like behavior in an open field was observed prior to seizure onset and was predictive of subsequent seizures. Immunohistochemical characterization of interneuron subtypes in GAD65^-/-^ mice showed a selective decrease in GABA and neuropeptide Y (NPY) levels and no change in calbindin (CLB) or calretinin (CLR) immunoreactivity in the hippocampus. Stem cells from the medial ganglionic eminence (MGE) were injected into the hippocampal hilus to restore GABAergic interneurons. One week after transplantation, MGE-transplanted mice demonstrated significant seizure resistance compared to sham surgical controls. The percent area of GFP^+^ MGE graft in the hippocampus correlated significantly with the increase in seizure latency. Our data indicate that impaired GABAergic neurotransmission can cause anxiety-like behavior and stress-induced seizures that can be rescued by MGE stem cell transplantation.

## Introduction

Psychiatric comorbidities are common among patients with epilepsy [1, 2]. Anxiety disorders are particularly prevalent, as children and adults with epilepsy experience anxiety at rates that are significantly increased compared to the general population and to those with other chronic diseases [3–6]. A United States population-based survey showed that 17% of children with epilepsy had been diagnosed with anxiety compared with 3% of those without epilepsy [7]. Additional studies of psychiatric comorbidity in the United States, Jordan, and Nigeria reported anxiety prevalence as high as 48.5% in children with epilepsy [8–10]. In adults with epilepsy, prevalence estimates for anxiety range from 11% to nearly 50% depending upon the population assessed and type of anxiety measure used [11]. In addition, the risk of anxiety is elevated even prior to seizure onset [2, 4]. The presence of psychiatric disorders may also be associated with the occurrence of cognitive complaints in patients with epilepsy [12, 13]. The increased rate of affective disturbance in epilepsy could be attributed to a common underlying neurobiological substrate, or to long term consequences of epilepsy and its treatment by antiepileptic drug therapy [6, 14]. Neurobiological mechanisms for the relationship between epilepsy and anxiety disorders remain poorly understood and systematic research in this area is yet lacking.

Biosynthesis of gamma-aminobutyric acid (GABA), the major inhibitory neurotransmitter in the mammalian brain, is dependent upon conversion of its precursor glutamate by glutamic acid decarboxylase (GAD). This enzyme exists in two isoforms within the brain, GAD67 and GAD65, each of which is thought to serve distinct functions given their differential location and regulation [15–17]. GAD67 is a cytosolic protein primarily localized to neuronal cell bodies where it catalyzes most basal GABA synthesis. GAD65 is a membrane-associated protein primarily located in neuron terminals and anchored in synaptic vesicles where it provides a dynamic buffer of GABA in situations of sudden demand such as fear or stress [18–20] and during seizures [21]. Mice deficient in GAD65 (GAD65^-/-^) on the non-obese diabetic (NOD/LtJ) background have significantly decreased basal GABA levels in the brain, develop spontaneous seizures as early as 3 months of age, and are susceptible to seizures precipitated by fear or mild stress [22]. Kash and his colleagues also found that 3 month-old GAD65^-/-^ mice on a C57BL/6 background exhibited anxiety-like behavior and reduced responsiveness to GABAergic anxiolytic drugs [23]. The presence of both recurrent seizures and an anxiety-like phenotype provides the opportunity to study the relationship between seizures and their comorbidities, and to investigate the evolution of epilepsy and its relationship to stress-related behavioral alterations in developing animals.

The medial ganglionic eminence (MGE), pallidal primordium located in the embryonic (E12.5—E13.5) ventral telencephalon, is an established rich source of GABAergic interneuron progenitor cells. These migrate to the striatum, cortex and hippocampus in the embryonic brain to provide inhibitory circuitry [24–26]. MGE-derived cells that are transplanted into the cortex have the capacity to migrate and functionally integrate into the postnatal brain parenchyma [27]. MGE progenitor cells grafted into cortex and hippocampus of adult mice can reduce both seizure activity and behavioral comorbidities [28–30]. Based primarily on these observations, it has been suggested that MGE-derived cells may be used to rescue deficits of brain inhibitory networks.

In the current study, we used young (< 8 weeks old) GAD65^-/-^ mice on a sensitive genetic background (NOD/LtJ) to investigate the association between seizures and anxiety, and the evolution of epilepsy over time. We further characterized the GABAergic interneuron phenotype and the relationship between spontaneous seizure onset and the development of the stress-related behavioral deficits. Finally, we investigated the ability of MGE progenitor cell transplantation to improve seizure susceptibility and behavioral deficits.

## Materials and methods

### Animals

All procedures were conducted in accordance with the National Institutes of Health Guidelines for the Care and Use of Laboratory Animals and approved by the Institutional Care and Use Committee of the Stanley Manne Children's Research Institute. Animals were kept under a 12-hour light/dark cycle. *GAD65 transgenic mice*: Four male and three female NOD 129X1-Gad2^*tm1Bae*^/J heterozygous mice (GAD65^+/-^) were obtained from Jackson Laboratory (#003653, Bar Harbor, ME). GAD65^-/-^ mice had been generated by inserting a neomycin resistance cassette into exon 1 of the *Gad65* gene downstream of the translation initiation start site. GAD65^-/-^, GAD65^+/-^ and GAD65^+/+^ mice were generated from heterozygous breeding and offspring were genotyped by PCR. PCR generated a 315 bp fragment from exon 1 of the *Gad65* gene using primer set oIMR 3457 5’- CATACGCAGACAGCACGTTT-3’ & oIMR 3473 5’- CAAACCCTAAACCACCCACA-3’ and a 530 bp *neo* gene using oIMR 3457 & oIMR 297 5’- CACGAGACTAGTGAGACGTG –3’. C57BL/6-Tg(CAG-EGFP)1Osb/J mice (Jackson Labs) were crossed to generate E13.5 embryos for GFP^+^ medial ganglionic eminence (MGE) cells.

### Anxiety-like behavior and susceptibility to stress-induced seizures

From 4 weeks to 8 weeks, GAD65^-/-^ (*n =* 20), GAD65^+/-^ (*n =* 8) and GAD65^+/+^ mice *(n =* 15) were placed individually into an unfamiliar, open arena for assessment of susceptibility to stress-induced seizures and anxiety-like behavior [31]. Using blinded observers, we found that 10-minute placement of mice in an open field arena consistently provoked seizures only in GAD65^-/-^ mice. Both male and female mice were used in each group. Exploratory behavior in an open field was stressful to these mice to result in all-or-none clearly identifiable motor seizures, and distinguished between the GAD-/- and wild-type mice well. We, therefore, used a 10-min open arena protocol to induce seizures in subsequent experiments.

Animals were placed into a 152.5 cm x 152.5 cm walled arena. On the floor of the arena, lines were drawn to mark off 25 (5x5) squares each sized 30.5 cm x 30.5 cm. During each 10-minute trial, the path taken, number of line crosses, and latency to and duration of seizures were recorded. The latency for mice that did not have seizures during the trial was defined as 600 sec. Behavioral seizures were observed and recorded independently by at least two observers who were blinded to genotype of the animals. Behavioral seizures were stereotyped and convulsive. All observed recorded events were sudden onset generalized clonic tonic seizures. Seizures followed a typical progression from falling to head and/or limb jerks and finally tonic stiffness lasting 30–60 seconds. Recovery occurred by one to four minutes. A separate group of “seizure-naïve” mice was not introduced to the open field until 8 weeks of age.

### Spatial learning and memory

To investigate the effects of GAD65 deficiency on spatial memory acquisition and retention, a subset of 4 week old GAD65^-/-^ (*n =* 11) and GAD65^+/+^ mice *(n =* 13), were trained and tested on a Barnes maze [32, 33]. The maze consisted of a flat circular platform elevated 105 cm above the ground and containing 20 equally spaced holes along its perimeter (platform diameter = 122 cm, hole diameter = 10 cm). Visual cues were placed on the walls surrounding the maze. During each trial, the animal was placed in the center of the platform and allowed to search for the hole leading to a darkened escape box. A trial was completed when (1) the animal entered the escape box or (2) 4 minutes had elapsed, at which point the animal was led to and placed in the escape box. The animal then remained in the escape box for 2 minutes for habituation. Animals were trained for four trials per day on four consecutive days. Consecutive trials were separated by at least 20 minutes. Escape latency (time taken to find and enter the escape box with front paws and trunk) was recorded for each trial. On day 11, seven days after the last training trial, a three-minute retention trial was performed to assess spatial memory retention.

### Immunohistochemistry

To characterize the effect of GAD deficiency on GABA and interneuron subpopulations, immunohistochemical staining for GABA, calbindin (CLB), calretinin (CLR), and neuropeptide Y (NPY) was performed on brain sections of 8-week-old GAD65^-/-^ mice (*n* = 3). In wild-type mice, GFP-labeled GAD65-positive interneurons can be subdivided according to the expression of NPY, cholecystokinin, CLB or CLR [34]. Mice were deeply anesthetized with sodium pentobarbital (80 mg/kg, *i*.*p*.) and perfused transcardially with 0.1M phosphate-buffered saline (PBS, pH 7.4) followed by 4% paraformaldehyde. Brains were removed, post-fixed overnight in the same solution and cryoprotected by immersion in 30% sucrose for at least 2 days. 40 μm coronal sections were cut on a freezing microtome and every 6th section was collected. The following primary antibodies were used: rabbit anti-GABA (Sigma, A2052; 1:500), rabbit anti-calbindin (CLB) (Swant, CB-38a; 1:1000), rabbit anti-caltetinin (CLR) (Swant, 7697; 1:2000), and rabbit anti-neuropeptide Y (NPY) (ImmunoStar, 22940; 1:2000). Brain sections were incubated with primary antibody at 4°C overnight. Following extensive washing, the sections were incubated in HRP-conjugated secondary antibody (Molecular probes, Invitrogen, Grand Island, NY) for 1 hour at room temperature. The signal was revealed with tyramide conjugated to Fluorescein using a TSA amplification kit (Molecular probes, Invitrogen, Grand Island, NY) according to the manufacturer’s instructions. Slide-mounted sections were examined on a LISA laser confocal microscope.

For quantification of GABA, NPY, CLR and CLB interneurons in the hippocampus, the percentage of immunopositive area was determined in GAD65^+/+^ and GAD65^-/-^ animals (*n =* 3/group). Six hippocampal sections per brain were selected for quantification. Analysis was completed using MetaMorph (v. 6.1, Universal Imaging Corp) by an investigator who was blind to the identity of the samples. Images were captured digitally at 20X magnification, converted to gray-scale and specific immunopositive areas were highlighted at a threshold set at a constant level for all specimens. An average percent area above the threshold was calculated per brain region.

### Stereotactic medial ganglionic eminence (MGE) cell transplantation

To investigate the potential for reversal of seizure and anxiety behavior, MGE stem cells were transplanted into a subset of GAD65^-/-^ mice (*n* = 10) that had experienced stress-induced seizures during open field testing on weeks 4 and 5. The MGE from transgenic mice expressing GFP were used to facilitate tracking of the migration and differentiation of grafted cells. Briefly, the MGE was dissected from transgenic E12.5-E13.5 mice into ice cold L-15 medium, and single cell suspensions (~1x10^5^ / μl in 1 μl) were prepared as previously described [35]. 24 hours after seizures on week 5, mice were anesthetized with sodium pentobarbital (80mg/kg, *i*.*p*.) and positioned in a mouse stereotaxic apparatus (Stoelting Co, IL) specially adapted for young mice. Following mechanical dissociation, the suspension was front-loaded into a 700 series Hamilton syringe (Reno, Nevada) and delivered over a period of 15 minutes. The injection needle was kept in place for additional 2 minutes. A total of 2 x 10^5^ MGE cells were injected into the hilus region of the dentate gyrus along the length of hippocampus at two separate sites at the following stereotaxic coordinates: (1) anterior-posterior (AP) -2 mm, medial-lateral (ML) -1 mm, dorsal-ventral (DV) -2.5 mm, and (2) AP -2.5 mm, ML -1.5 mm, DV -2.8 mm. Sham-operated controls (*n* = 6) were injected with same volume of culture medium (1 μl per site). All animals underwent open-field testing once weekly for three consecutive weeks following surgery (6, 7 & 8 weeks old). One day after open-field testing at 8 weeks old, mice were deeply anesthetized with 80 mg/kg i.p. pentobarbital, perfused transcardially with 4% paraformaldehyde, and their brains harvested for quantification of MGE cell integration.

### Quantification of GFP^+^ MGE cell integration

40 μm coronal sections of brain tissue were cut on a freezing microtome and every 6th section containing hippocampus was collected. At least 6 hippocampal sections per brain were analyzed. The brain of each MGE-transplanted GAD65^-/-^ mouse was scored on the following parameters: (A) Position of injection site (1 = reached hippocampus but not hilus; 2 = reached hippocampus and hilus; (B) Distance of GFP^+^ cell migration (0.5 = <1mm; 1.0 = 1–1.2mm; 1.5 = 1.3–1.5mm; 2 = >1.6mm, (C) Number of neurons with mature morphology (0 = no neurons with mature morphology; 1 = 1–40 neurons with mature morphology; 2 = more than 40 neurons with mature morphology, (D) Percentage of GFP^+^ area (0.5 = <5%; 1 = 5–10%; 1.5 = 11–19%; 2 = >20%), (E) Specific migration pattern (0 = no GFP^+^ cells migrated to hilus; 1 = 1–25 GFP^+^ cells migrated; 2 ≥ 25 GFP^+^ cells migrated). The scores on parameters A-E were subsequently combined into a total integration score.

### Statistical analysis

A Student’s t-test was performed to compare the difference in immunohistochemical staining between GAD65^+/+^ and GAD65^-/-^mice, and to compare seizure latency between seizure-experienced versus seizure-naïve animals at 8 weeks. A one-way analysis of variance (ANOVA) with post-hoc Tukey’s multiple comparison was used to compare changes in latency to seizures over time and to compare differences in behavior in the open field test and Barnes maze. Fisher's Exact Test was used to compare percentages of mice with stress-induced seizures at each week. Pearson’s chi-square test was used to test the correlation between seizure latency and the total score of each MGE transplanted mouse, as well as between seizure latency and the percentage of GFP-positive area. If not otherwise provided, denoted numbers (*n*) refer to number of animals. Data are expressed as mean ± SEM. The significance level was set to *p*<0.05.

## Results

### Recurrent stress-induced seizures lead to an epileptic state in GAD65 ^-/-^ mice

**Fig 1:**
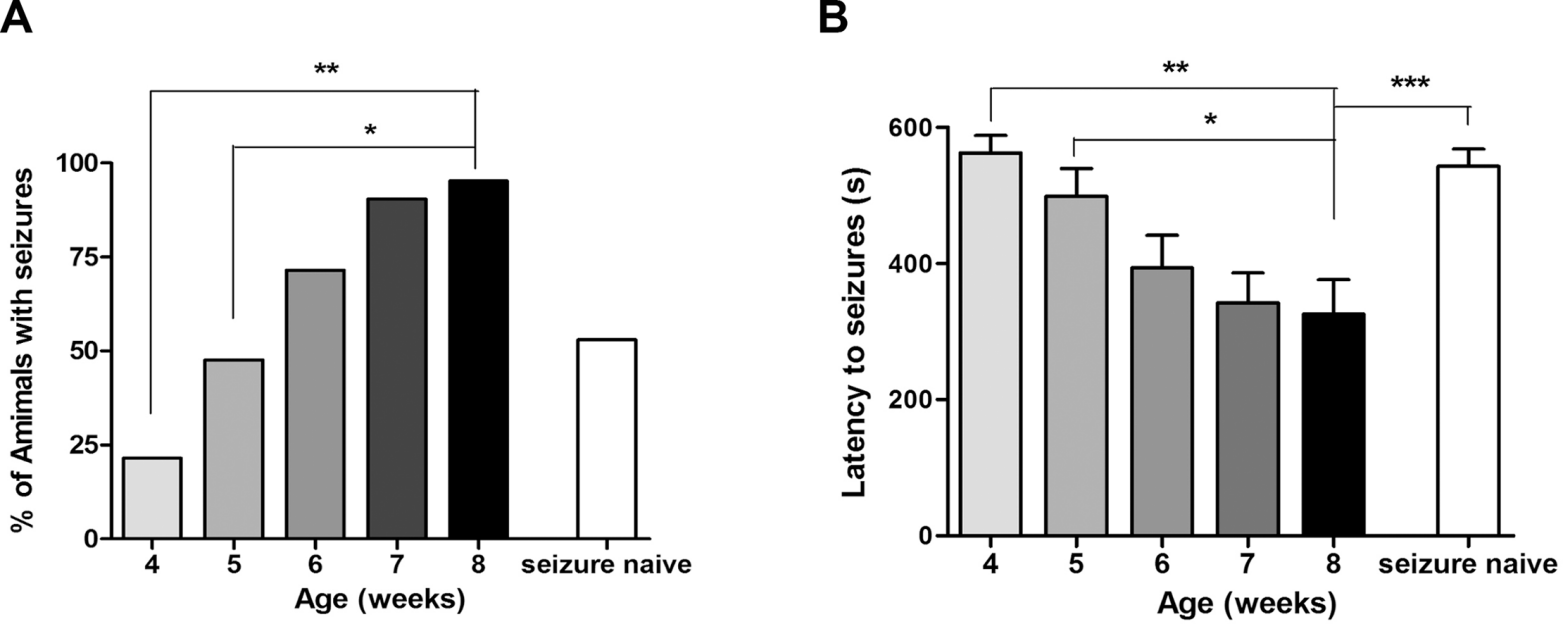
Recurrent stress-induced seizures lead to an epileptic state in GAD65 ^-/-^ mice. **A.** Percentage of animals that experienced stress-induced seizures by week. At 4 weeks of age, 21% of GAD65 ^-/-^ mice (*n* = 20) exhibited stress-induce seizures during open field testing. By 8 weeks of age, this number increased to 95%. The differences between weeks 4 and 5 compared to week 8 were significant (Fisher's Exact Test, 4 vs. 8 weeks: *p* < 0.01, 5 vs. 8 weeks: *p* < 0.05). In contrast to animals that experienced the weekly stress of open field testing, only 53% of seizure-naïve animals (*n* = 12) exhibited seizures during open field testing at week 8. **B.** Seizure latency by week. The latency to seizures decreased with each subsequent week in GAD65^-/-^ mice (*n* = 20) (One-way ANOVA, *p*<0.05). Latency was significantly greater at 4 and 5 weeks compared to at 8 weeks (one-way ANOVA with Tukey’s post-hoc multiple comparison test, 4 vs. 8 weeks: *p*< 0.01, 5 vs. 8 weeks: *p*< 0.05). Mice that experienced prior stress-induced seizures had a significantly shorter latency (253 ± 48 sec) to seizures than the group that experienced their first seizures at 8 weeks (492s ± 41 sec) (*t*-test, *p*<0.005).

At 4 weeks of age, placement in an open field provoked seizures in 21% of GAD65^-/-^ mice. By 8 weeks, 100% of GAD65^-/-^ mice experienced a seizure at least once during testing from weeks 4–8. At 8 weeks of age, 95% of GAD65^-/-^ mice exhibited seizures if they were exposed to the weekly stress of open field testing (Fig 1A). In contrast, only 53% of 8-week-old seizure-naïve GAD65^-/-^ mice exhibited seizures when they were placed in the open field for the first time. Also, mice that experienced prior stress-induced seizures had a significantly shorter latency to seizures than the group that experienced their first induced seizures at 8 weeks (Fig 1B). Thus, the heightened seizure susceptibility observed in 8 week-old GAD65^-/-^ mice cannot be attributed entirely to age, but appears to be, at least in part, due to the cumulative effects of recurrent seizures. The open field failed to induce seizures in any GAD65^+/+^ or GAD65^+/-^ mouse at any point during testing. Latency to seizures in GAD65^-/-^ mice decreased on each successive week of testing. There was also a significant decrease in latency in week 8 compared to weeks 4 or 5 (Fig 1B).

### Anxiety-like behavior in GAD65 ^-/-^ mice

**Fig 2:**
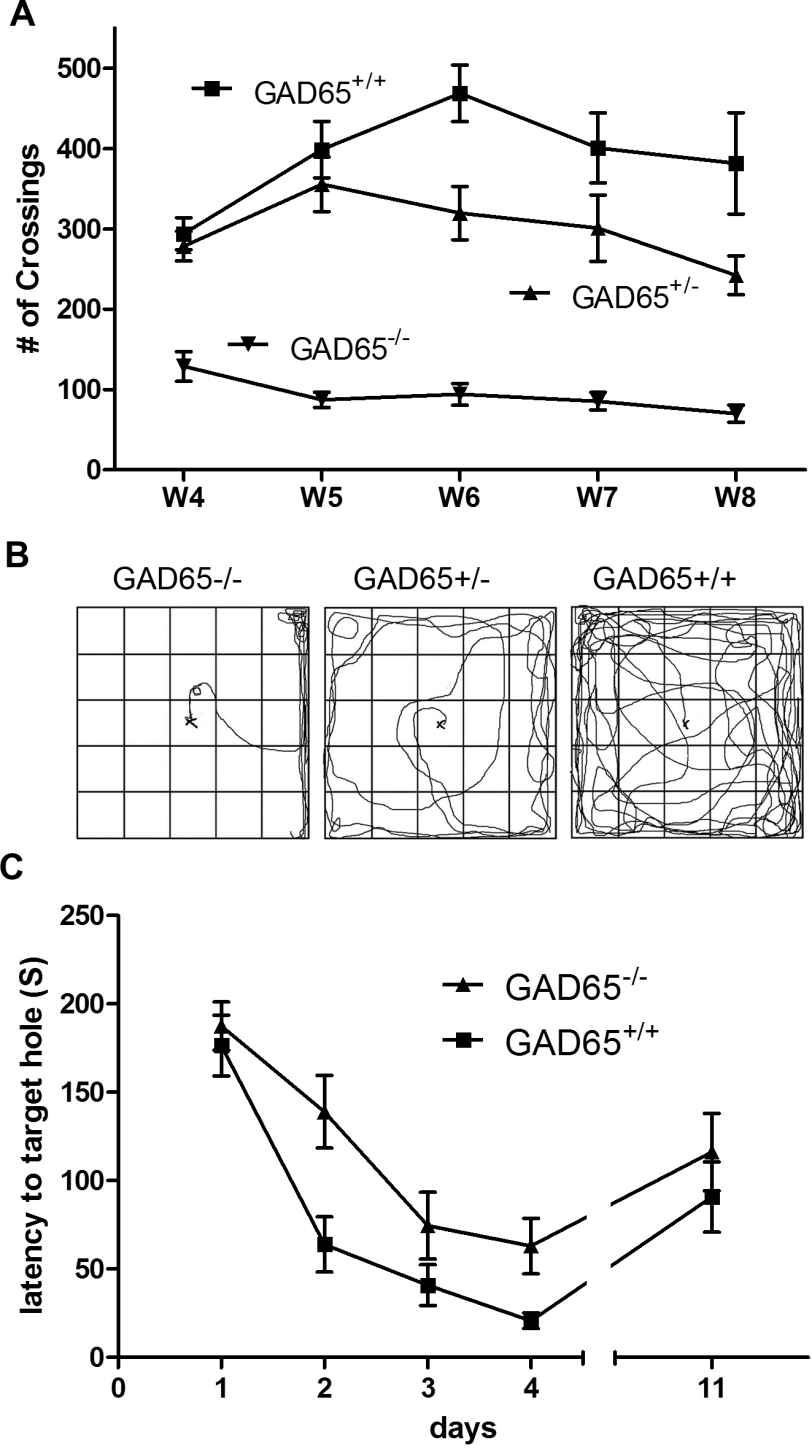
Impaired behavior in GAD65 ^-/-^ mice: GAD65 ^-/-^ mice (*n = 20*) exhibited marked behavioral impairments compared to GAD65 ^+/+^ *(n = 15*) and GAD65^+/-^ mice (*n = 8*). **A.** Open field line cross frequency: During open field testing, GAD65^-/-^ mice explored the arena significantly less, quantified as number of line crossings, than their GAD65^+/+^ littermates (4 weeks: 97 ± 47 vs. 273 ± 72; 5 weeks: 87 ± 42 vs. 355 ± 104; 6 weeks: 94 ± 61 vs. 430 ± 88; 7 weeks: 86 ± 48 vs.345 ± 68; 8 weeks: 70 ± 48 vs. 275 ± 62; t-test: *p*<0.01). GAD65^-/-^ mice explored significantly less than GAD65^+/+^ mice whether or not they experienced seizures during open field testing (*t*-test: *p*<0.0001). GAD65^+/-^ heterozygotes also explored significantly less than GAD65^+/+^ mice, though the difference was not as pronounced (*t*-test: *p*<0.05). Decreased exploratory behavior was observed in GAD65^-/-^ mice prior to the first occurrence of stress-induced seizures and was predictive of seizure occurrence in individual animals in subsequent weeks. **B.** Open field path tracings: GAD65 ^+/+^ mice explored all areas of the arena without hesitance and maintained a high level of activity during each trial. In contrast, GAD65 ^-/-^ mice engaged in minimal exploration of the arena and tended to stay near the walls rather than entering the center. GAD65 ^+/-^ mice explored the arena to a greater extent than GAD65^-/-^mice, but did exhibit a tendency to stay near the walls and corners. **C.** Barnes maze assessment of visual-spatial learning: Both GAD65^+/+^ and GAD65^-/-^ mice exhibited learning over the 4 day acquisition period with decreasing average escape latencies from day 1 to day 4 (t-test, day1 vs. day4, GAD65^+/+^: p < 0.0001; GAD65^-/-^: p < 0.0001). However, GAD65^-/-^ mice exhibited a significant retardation in learning compared to GAD65^+/+^ mice (one-way ANOVA day1-4: *p*< 0.0001). There was no significant difference in retention of learning between these two groups during the probe test (day11; *t-*test: *p*>0.05).

The open-field test was used to evaluate anxiety-like behavior in GAD65 ^-/-^, GAD65 ^+/-^ and GAD65^+/+^ mice. When placed in a novel environment such as an open field, rodents typically tend to stay near the periphery initially (thigmotaxis), only later exploring the central parts of the arena. Anxious animals remain around the periphery and/or remain stationary for longer periods of time [14, 36]. Over 4 weeks of observation, both GAD65 ^-/-^ and GAD65 ^+/-^ mice consistently exhibited marked impairments in exploratory behavior compared to GAD65^+/+^ littermates, quantified as the number of line crosses made (repeated measures *t*-tests, *p*<0.01, *p*<0.05, respectively) (Fig 2A). This profound deficit in exploratory behavior was present in 100% of GAD65^-/-^ mice at 4 weeks of age even in those mice that did not experience seizures during open-field testing. GAD65^-/-^ mice tended to remain longer in a corner of the open field and walked slowly with persistent thigmotaxis (Fig 2B). Prior to seizure onset, GAD65^-/-^ mice exhibited cautious slow movements and freezing cessation of activity. After seizures, the mice underwent a brief period of recovery followed by hyperactivity and running. Both the line count data and pathway tracings show that GAD65^-/-^ mice were more hesitant and fearful in exploring the open field than wild type controls. Interestingly, the exploratory behavior of heterozygous (GAD65 ^+/-^) mice was between that of wild type (GAD65 ^+/+^) and homozygous (GAD65 ^-/-^) mice (Fig 2A & 2B).

### Impaired spatial learning acquisition in GAD65 ^-/-^ mice

The Barnes maze was used to assess spatial learning and memory in GAD65^+/+^ and GAD65^-/-^ mice. Both GAD65^+/+^ and GAD65^-/-^ mice demonstrated significant spatial learning on the Barnes maze over the 4-day acquisition period, with decreasing average escape latencies from day 1 to day 4 (*t*-tests, day1 vs. day4, GAD65^+/+^ *p*< 0.0001; GAD65^-/-^ *p* < 0.0001). However, GAD65^-/-^ mice exhibited significantly increased escape latencies compared to GAD65^+/+^ animals during the training phase (one-way ANOVA day 1–4: *p*< 0.0001) (Fig 2C). There was no difference between the two groups on probe trial testing at the end of the acquisition period.

### Reduced expression of GABA and NPY in GAD65^-/-^ mice

**Fig 3:**
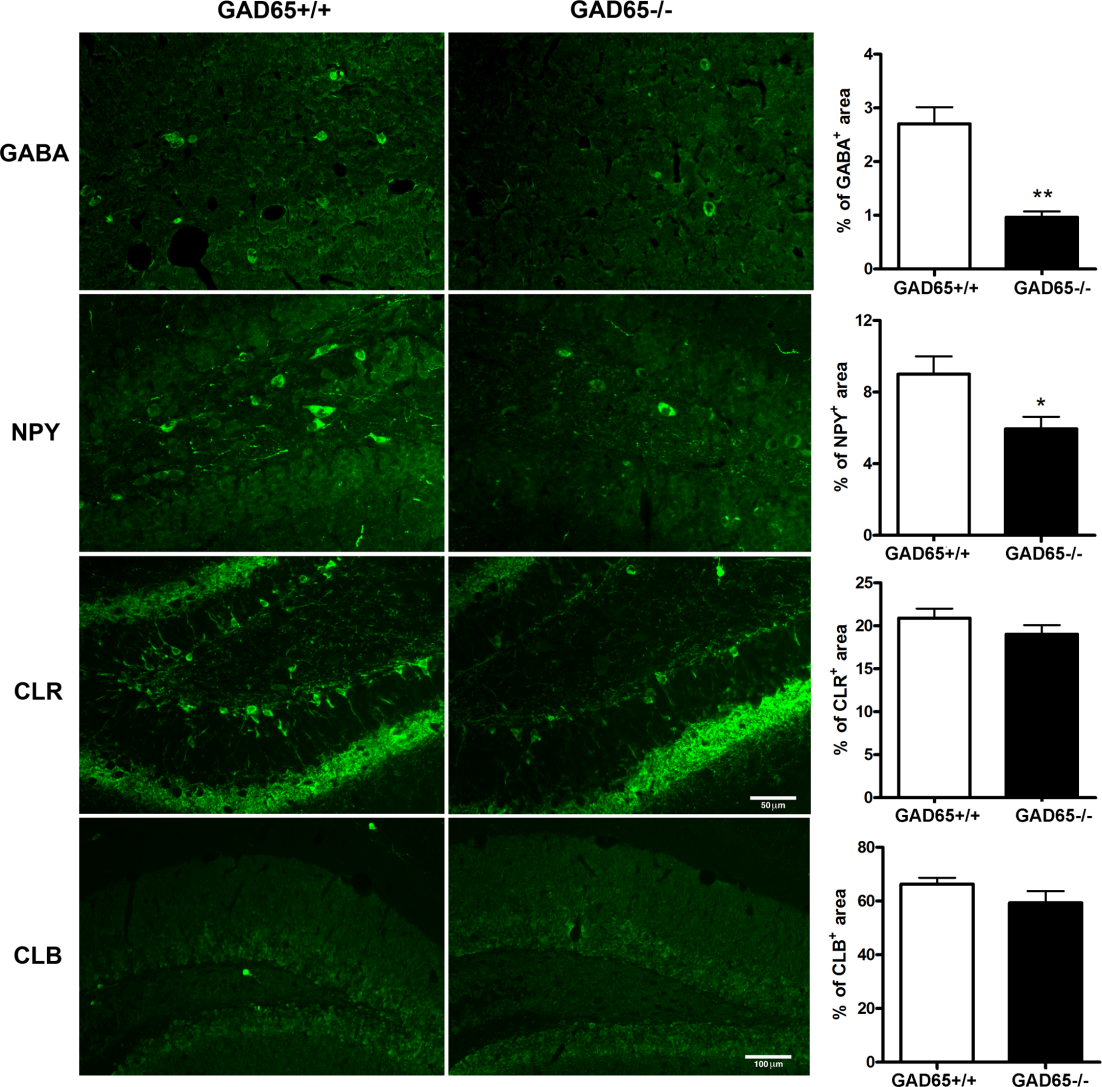
Subtypes of interneuron changes in the GAD65^-/-^ mice: GABA and neuropeptide Y (NPY) expression were significantly reduced in the hippocampi of GAD65^-/-^ mice compared to GAD65^+/+^ mice, (GABA: 2.7 ± 0.31 vs. 0.97 ± 0.11, p<0.001; NPY 9.0 ± 0.99 vs. 6.0 ± 0.68, p<0.02). No significant differences in CLB or CLR expression were noted. The scale bar of 50μm is the same for all GABA, NPY and CLR photomicrographs; for CLB, the scale bar = 100μm.

We examined the expression of the inhibitory neurotransmitter GABA and the interneuron markers NPY, CLR and CLB in hippocampal sections by immunohistochemistry to determine whether GAD65 deficiency reduced the expression of interneuron markers in young mice. Significantly fewer GABA-positive cells were present in the hippocampi of GAD65^**-/-**^ mice compared to wild-type controls (*p*<0.001) (Fig 3). GAD65^-/-^ mice also showed significantly lower NPY expression (*p*<0.05). There were no significant differences in CLB or CLR expression in the hippocampus.

### MGE transplantation transiently reduces seizure susceptibility

**Fig 4:**
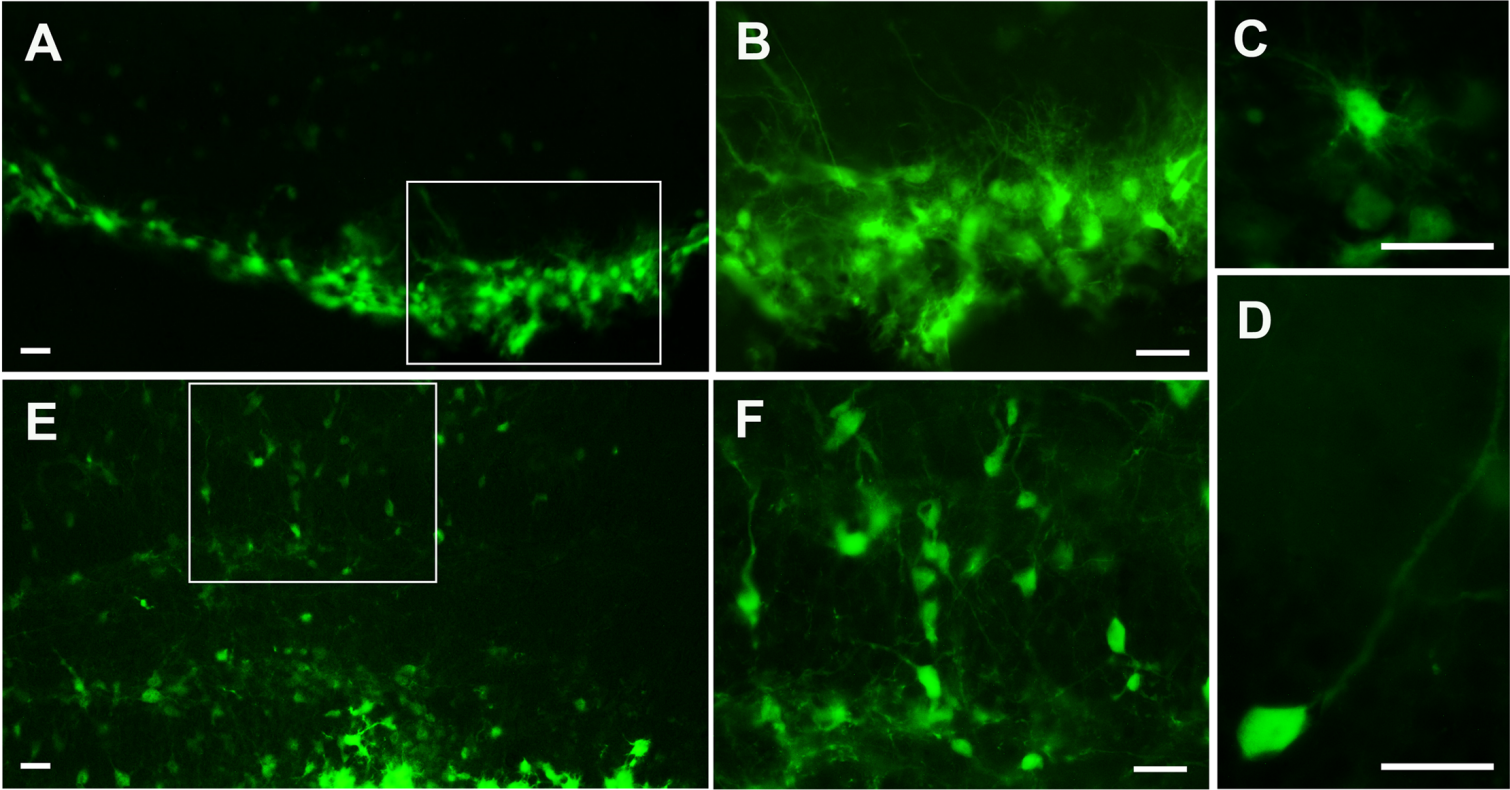
Survival and morphology of transplanted medial ganglionic eminence (MGE) cells in GAD65^-/-^ mice. 3 weeks after transplantation, GFP^+^ MGE cells showed high survival and migrated extensively within the hippocampus and especially the hilus (A, E). B and F are higher magnificent from A and E. The majority of grafted cells exhibited multiple processes (C), while some demonstrated a thin and longer axon-like process (D) or bipolar or multipolar morphology. Scar bar = 50um.

**Fig 5:**
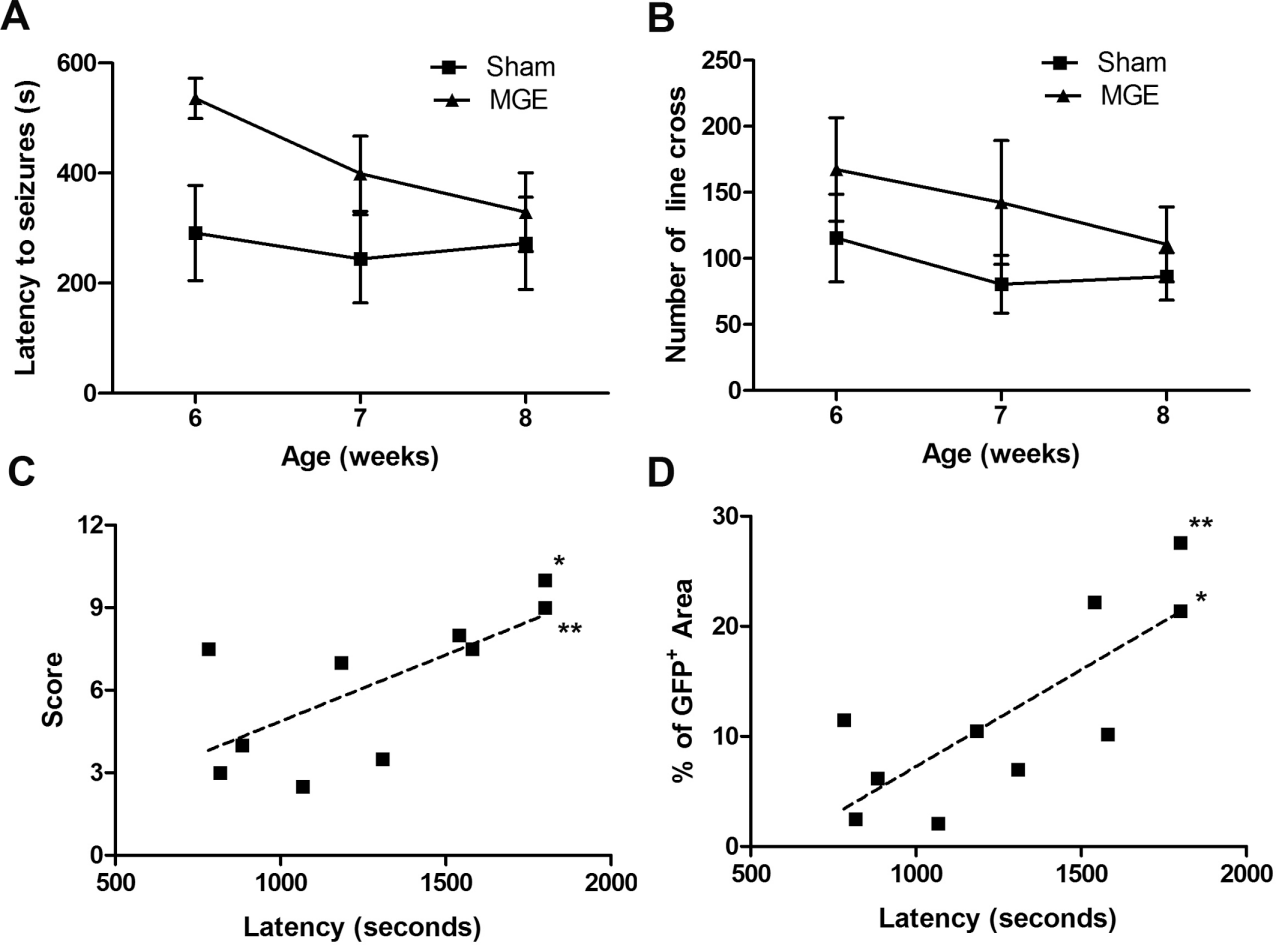
Effects of medial ganglionic eminence (MGE) transplantation on seizure susceptibility and behavior in GAD65^-/-^ mice. **A.** Latency to seizures 1–3 weeks after transplantation of MGE cells. One week after MGE cell transplantation (6 weeks of age), there was a significant increase in latency to stress-induced seizures in GAD65^-/-^ mice (transplanted *n* = 10; sham *n* = 6; *t-*test, *p*<0.01,). There was no significant difference in seizure latency by 2 (7 weeks of age) or 3 weeks (8 weeks of age) after transplantation. (2 and 3 weeks: *p*>0.05). Error bar = Mean ±SD. **B.** Open field exploration 1–3 weeks after transplantation of MGE cells. GAD65^-/-^ mice that received MGE cell transplants exhibited a trend toward increased frequency of line crosses during open field testing, though the difference was not statistically significant (repeated measure t-test, *p* = 0.054). Error bar = Mean ±SD. **C, D.** Latency to stress-induced seizures (sum of latencies at weeks 1–3 after transplantation) in mice that received MGE cell transplants was significantly correlated with total integration score (C, *r =* 0.025) and percentage area of GFP+ within the hippocampus (D, *r* = 0.007), suggesting a relationship between the success of the graft and decreased seizure susceptibility. Notably, the two mice that remained seizure-free 3 weeks after MGE transplantation (* and **) had both the highest total integration score and percentage area of GFP+ within the hippocampus.

MGE stem cell transplantation was performed to investigate whether restoration of hippocampal GABA would decrease seizure susceptibility and behavioral deficits in GAD65^-/-^ mice. The GFP^+^ MGE cells survived and migrated into the hilus of the hippocampus in transplanted animals to a varying degree (Fig 4). The latency to seizures in MGE-transplanted mice was correlated with a total integration score (r = 0.025) and percentage of GFP^+^ area in the brain (r = 0.007) (Fig 5C & 5D). MGE-transplanted mice exhibited decreased seizure susceptibility compared to sham operated controls. At 6 weeks of age, one week after MGE-transplantation, MGE-transplanted mice had an increased latency to stress-induced seizures compared to controls (*t*-test *p*<0.01) (Fig 5A). Also, at 6 weeks of age, only 30% (3/10) MGE-transplanted mice exhibited stress-induced seizures while 83% (5/6) of sham-operated controls experienced seizures during the open-field test (Table 1). All sham-operated control mice exhibited stress-induced seizures by 8 weeks of age, 3 weeks after surgery. Notably, 2/10 MGE-transplanted mice remained seizure-free at 8 weeks of age. These two MGE-transplanted seizure-free mice were found to have both the highest integration scores and greatest percent area of GFP^+^ MGE cells (**Fig 5C & 5D**, * & **). Exploratory behavior was not significantly different between MGE-transplanted and control groups (**Fig 5B**).

**Table 1:** Comparison of resistance to stress-induced seizures.

	*Seizure induction (%)*					*No seizure induced by the end of 8 weeks*
Age (week)	4	5	6	7	8	
No surgery	21	48	71	90	95	0/20
Sham			83	83	83	0/6
MGE			30	50	80	2/10

## Discussion

### Anxiety and seizures in GAD65-deficient mice

In the current study, we observed that GAD65 knockout mice were highly susceptible to stress-induced seizures as early as 4 weeks of age and that anxiety-like behavior was evident prior to the onset of seizures. While open-field testing provoked seizures in less than 25% of GAD65^-/-^ mice at 4 weeks of age, 100% of GAD65^-/-^ mice exhibited decreased exploratory activity and increased thigmotaxis in the open field at that time. All GAD65^-/-^ mice eventually developed stress-induced seizures during open-field testing in subsequent weeks, suggesting that decreased exploratory behavior was predictive of seizure susceptibility. The relationship between seizures and anxiety, however, remains correlative rather than clearly causal. Additionally, although we did not observe apparent gender differences in behavioral testing, gender has been reported to influence responses to early-life stress [37] and epilepsy [38], and remains an unstudied aspect in the current study.

Several studies examining patients with both acquired and genetic epilepsies have similarly identified the presence of psychiatric disturbance prior to the onset of seizures [39–41]. Psychiatric disorders often pre-date epilepsy and, in fact, present a risk factor for the later development of epilepsy [42]. Placement of an animal in an open field was sufficient to cause seizures in nearly all GAD65^-/-^ mice if they had been subjected to repeated stress and seizures while only about half of seizure-naïve 8 week-old GAD65^-/-^ exhibited seizures under stress. Recurrent stress-induced seizures were associated with a progressive increase in seizure susceptibility over time in these GAD65^-/-^ mice showing the ability of seizures to promote secondary epileptogenesis. Future studies of potential biomarkers and anatomical or genetic substrates of epileptogenesis broadly could both support and provide more mechanistic data on this relationship [43, 44].

GAD65^-/-^ mice also showed significant deficits in spatial learning during the acquisition phase of the Barnes maze at 4 weeks of age. Previous studies have provided evidence that the GABAergic system may play a role in spatial reference memory [45, 46]. Similar impairments in learning and memory have been observed in several rodent models of epilepsy [47–50]. Likewise, patients with temporal lobe epilepsy often report difficulties learning new information while retention of learned information is less affected [51]. Interestingly, anxiety may contribute to cognitive deficits in patients with epilepsy. In a multivariable regression analysis of factors associated with memory complaints among Hong Kong adults with epilepsy, anxiety was the most important independent predictor of memory complaints [6, 12].

### Rescue of GABA-deficient phenotype by MGE cell transplantation

We demonstrated that GABA-rich MGE neuroprogenitor cells survive in the brain of GAD65^-/-^ mice after direct injection into the hippocampus and that transplantation decreased susceptibility to stress-induced seizures in these animals. GABA, the major inhibitory neurotransmitter in the mammalian central nervous system, plays an important role in the pathogenesis of both epilepsy and anxiety. In human epilepsy and in animal models of genetic epilepsy, reduced GABAergic inhibition in the brain results in excessive excitation and seizures. For instance, mutations in the SCN1A gene, encoding voltage-gated sodium channels, cause a selective decrease in excitability of GABAergic interneurons that lead to the severe epilepsy of Dravet syndrome [52–55]. In addition, the loss of subsets of GABAergic neurons is one of the pathological hallmarks of chronic temporal lobe epilepsy [56–58]. Failure of the GABA system has also been implicated in anxiety disorders, and anxiety attributed to a decrease in GABA within the hippocampus and amygdala [59]. Specifically, GABAergic neurotransmission in the amygdala is a promising candidate for modulation of anxiety-related responses [60]. Infusion of GABA or GABA receptor agonists into the amygdala decreases anxiety responses in several animal species while infusions of GABA antagonists tend to have anxiogenic effects [61, 62]. In contrast, selective deactivation of GAD expression in the amygdala leads to loss of the anxiolytic effect of benzodiazepines [63]. In humans, low extracellular GABA levels in plasma [64, 65] and cerebral spinal fluid [66, 67] have been identified in patients with mood and anxiety disorders. Drugs that agonize GABA_A_ receptors increase the threshold for seizures and also control anxiety [68]. In contrast, drugs that block GABA_A_ receptors are proconvulsant and induce symptoms of anxiety [69]. Thus, the decrease in stress-induced seizures by MGE cell transplantation is further supportive of this approach to treat broadly, disorders that arise from a deficiency of GABA by increasing available GABA.

It is worth noting that the animals in this study that exhibited the highest GFP-positive area were the most seizure-resistant during the three weeks of open-field testing (**Fig 5C & 5D**). The transient effect of MGE transplantation could have been related to the limited long-term survival of the implanted cells. Further work will be needed to investigate the long-term survival of MGE transplants and its relation to behavior. We provide additional proof of concept that transplantation therapy has the potential to rescue deficits of brain inhibitory networks and restore the imbalance of inhibition-excitation that may be the core problem in multiple forms of epilepsy. Although production of a comparable and safe MGE-like human stem cell line will ultimately be necessary before translation to the clinic, our results are an encouraging step toward using inhibitory neurons for brain repair in children with severe forms of epilepsy. This would be an important development because prolonged drug treatment can have unwanted cognitive or neurobehavioral side effects, current medications that systemically potentiate GABA-medicated inhibition are incompletely effective at suppressing seizures, and epilepsy remains refractory to medical treatment in nearly one-third of affected individuals[70].

### Potential contribution of neuropeptide Y

The GAD65^-/-^ mice in our study showed a greater than 50% decrease in GABA levels, and a nearly 50% decrease in NPY expression in the hippocampus, with no change in the calcium-binding proteins calbindin or calretinin. We cannot say why NPY was selectively diminished, but it is well established that different inhibitory neuronal subsets can have varied roles in physiological and pathophysiological activity of hippocampal and neocortical circuits [71–73]. For example, increased firing of perisomatic GABAergic synapses that arise from specific interneuron subsets has been hypothesized to initiate seizure-like events [71], and optogenetic activation of parvalbumin and somatostatin neurons in the hippocampus were recently reported to trigger seizure-like events [72].

We chose to examine NPY for its reported role in stress models and possible promotion of sustained seizures. Notably, NPY could play a significant role in stress and anxiety independent of GABA [74, 75]. Both intracerebroventricular administration of NPY [76] and genetic overexpression of NPY in the hippocampus [77] decreased anxiety levels in rats, while NPY knockout mice displayed increased anxiety in an open field [78]. Additionally, NPY knockout mice, compared to wild-type, exhibited more frequent EEG seizures that were longer in duration and progressed to status epilepticus and death within an hour after kainic acid administration, and further, NPY pretreatment prevented the mortality [79]. These findings suggest that the loss of NPY in GAD65^-/-^ mice could be more than just indicative of a loss of GABAergic interneurons, and that decreased NPY signaling could be an additional contributing factor to the anxiety that preceded seizures and to the seizures induced by stress in this model.

In conclusion, our findings indicated that GAD65^-/-^ mice are a useful tool to model the pathogenesis and treatment of epilepsy and psychiatric comorbidities commonly associated with epilepsies and epilepsy syndromes. We demonstrated increased anxiety-like behavior in GAD65^-/-^ mice that is present prior to the onset of stress-induced seizures. This finding points to shared causal antecedents in the behavioral and epilepsy phenotypes and indicates that anxiety could be an “essential co-morbidity” in epilepsy. Such shared causality could in part explain the high prevalence of anxiety disorders in individuals with epilepsy. We also provided evidence for epileptogenesis in GAD65^-/-^ mice that was most likely attributable to impaired GABAergic neurotransmission, and could be rescued by transplantation of MGE cells that differentiate into GABA-secreting neurons. This type of transplantation therapy has been shown to exert seizure resistance in multiple animal models, and our data further support its promising potential.
